# Caplacizumab for immune thrombotic thrombocytopenic purpura: real-world multicenter data

**DOI:** 10.3389/fmed.2023.1226114

**Published:** 2023-10-11

**Authors:** Eleni Gavriilaki, Emmanuel Nikolousis, Eudoxia-Evaggelia Koravou, Sotiria Dimou-Besikli, Charalampos Kartsios, Anna Papakonstantinou, Anastasia Mpanti, Charalampos Pontikoglou, Christina Kalpadaki, Aikaterini Bitsani, Ilianna Tassi, Tasoula Touloumenidou, Thomas Chatziconstantinou, Maria Papathanasiou, Antonia Syrigou, Eleutheria Ztriva, Georgia Kaiafa, Evdokia Mandala, Zois Mellios, Dimitrios Karakasis, Alexandra Kourakli, Argiris Symeonidis, Eleni Kapsali, Helen H. Papadaki, Chrysavgi Lalayanni, Ioanna Sakellari

**Affiliations:** ^1^BMT Unit - Department of Hematology, G. Papanicolaou Hospital, Thessaloniki, Greece; ^2^Department of Haematology, Athens Medical Center, Athens, Greece; ^3^Heartlands Hospital, Birmingham, United Kingdom; ^4^Medical School, Aristotle University of Thessaloniki, Thessaloniki, Greece; ^5^Department of Hematology, Papageorgiou Hospital, Thessaloniki, Greece; ^6^Department of Hematology, University of Crete School of Medicine, Crete, Greece; ^7^First Department of Internal Medicine, LAIKO General Hospital, Athens, Greece; ^8^Department of Hematology, University Hospital, Ioannina, Greece; ^9^1st Medical Propaedeutic Department of Internal Medicine, Aristotle University of Thessaloniki, Thessaloniki, Greece; ^10^4th Department of Internal Medicine, Aristotle University of Thessaloniki, Thessaloniki, Greece; ^11^Department of Hematology, Evangelismos Hospital, Athens, Greece; ^12^Division of Hematology, Department of Internal Medicine, University Hospital of Patras, Patras, Greece

**Keywords:** caplacizumab, thrombotic thrombocytopenic purpura, plasma exchange, ADAMTS13, multicenter real-world study

## Abstract

Given the limited real-world data of caplacizumab, our multicenter real-world study was designed to assess the safety and efficacy of caplacizumab in immune thrombotic thrombocytopenic pupura (iTTP), compared to historic controls. We have studied 70 patients: 23 in the caplacizumab and 47 in the historic control group. Plasma exchange was applied in all episodes except for two patients that denied plasma exchange. Rituximab as first-line treatment was more common in the caplacizumab group compared to historic control. Caplacizumab (10 mg daily) was given at a median on day 7 (1–43) from initial diagnosis for 32 (6–47) dosages. In the caplacizumab group, a median of 12 (8–23) patients required plasma exchange sessions versus 14 (6–32) in the control group. Caplacizumab administration did not produce any grade 3 complications or major hemorrhagic events. After a median of 19.0 (2.6–320) months since the iTTP diagnosis, 5 deaths occurred (4 in the control group and 1 in the caplacizumab group, *p* = 0.310). Caplacizumab patients achieved early platelet normalization and ADAMTS13 activity normalization at the end of treatment. Relapse was observed only in 2/23 (9%) caplacizumab patients, compared to 29/47 (62%) historic controls (*p* < 0.001). Overall, caplacizumab is safe and effective in treating iTTP, including cases refractory to plasma exchange, re-administration, and cases without previous plasma exchange treatment. No major hemorrhagic events were observed. Cessation of dosing guided by ADAMTS13 has ensured a low relapse rate.

## 1. Introduction

Almost one century ago, in 1924, Thrombotic Thrombocytopenic Purpura (TTP) was clinically described for first time by Eli Moschcowitz. In particular, a 16-year-old girl was diagnosed with a fatal thrombotic microangiopathy (TMA) syndrome, characterized by fever, transient focal neurologic symptoms, severe thrombocytopenia, and microangiopathic hemolytic anemia. These findings were linked to the presence of autopsy-defined systemic visceral microthrombosis of the terminal arterioles and capillaries (1). During the past 20 years, acquired or immune TTP (aTTP or iTTP) has been transformed from a clinical diagnosis of exclusion into a fully-described pathophysiologic diagnosis, based on specific clinical and laboratory features (2). It is now a well-established medical emergency requiring a rapid diagnosis and management. Death may occur usually during the acute phase, of the disease, resulting from uncontrolled formation of microvascular thrombi (3). Severe ADAMTS13 deficiency (<10%) is both, sensitive and specific for the diagnosis of TTP (4). Despite the advances in treatment options for TTP, there are still limited high quality data to inform clinicians regarding the recently introduced targeted type of treatment.

Established treatment approaches—plasma exchange and immunosuppression—replenish functional ADAMTS13 enzyme, but do not adequately address microvascular thrombosis (5). Caplacizumab represents the first drug to receive a regulatory approval for the treatment of iTTP. Caplacizumab, an anti–von Willebrand factor humanized, bivalent variable-domain-only immunoglobulin fragment, inhibits interaction between von Willebrand factor multimers and platelets (6). The drug demonstrated efficacy and safety in the placebo-controlled phase- 2 TITAN and phase-3 HERCULES studies. Both studies concluded that caplacizumab treatment is generally well tolerated, hastens platelet recovery, and reduces the recurrence rates. Relapses, however, were more common among caplacizumab- treated patients in both studies (7, 8). Despite the safety and efficacy of caplacizumab, several questions remained unanswered by these randomized clinical trials and the subsequent analyses (9). Caplacizumab has also been used in pregnancy (10) and for treatment of pediatric patients (11). Data from clinical trials also suggest that the benefit of caplacizumab is greatest when it is given earlier in the course of disease (8), although this is not always feasible in clinical practice.

Given the limited real-world data of caplacizumab, our multicenter real-world study was designed to assess the safety and efficacy of caplacizumab, compared to historic controls.

## 2. Materials and methods

### 2.1. Patients

We recorded clinicobiological data from consecutive adult patients (≥18 years of age), diagnosed with iTTP in the last 10 years (2011–2022). Diagnosis was based on clinical presentation (anemia, thrombocytopenia, and microangiopathic hemolytic anemia) and was confirmed with measurement of plasma ADAMTS13 activity by commercially available ELISA kit (Technozym), indicating severe ADAMTS13 deficiency (<10%). Patients were treated according to current guidelines, as implemented by their treating physicians. International guidelines were implemented in all centers since 2020 (6). In the era of caplacizumab, ADAMTS13 activity levels were available within 48 hours from diagnosis, and they were also used to guide caplacizumab dosing, which was administered until ADAMTS13 activity was raised above 10% (defined as ADAMTS13 normalization). Both, caplacizumab and historic control patients have been continuously monitored until data cut-off (October 2022). TTP event occurring more than 30 days after the end of daily plasma exchanges, was referred to as relapses. Exacerbations were defined as recurrent thrombocytopenia within 30 days after the end of daily plasma exchanges that required reinitiation of daily exchanges. Major or minor bleeding events were determined by treating physicians. Patients provided written informed consent to participate in the study which was conducted according to the Helsinki Declaration.

### 2.2. Study design

We conducted this comparative real-world multicenter study at 11 Hematology Departments (10 based in Greece and one in the United Kingdom). Patient records were documented retrospectively in a predefined CRF format. Standard of Care (SOC) treatment was implemented according to each center’s protocol. The majority of the participating centers administered steroids, as methylprednisolone 1mg/kg, along with daily plasma exchange treatment with subsequent tapering of steroids and frequency of plasma exchange. Most centers also administered rituximab weekly x 4 doses. The historical control group received rituximab based on physician’s decisions, mostly in refractory/relapsed patients or patients with severe presentation (i.e., neurological manifestations). Membrane filtration was used in six centers, while centrifugal plasma exchange in five centers. Relapse was defined as in deterioration after 30 days in remission.

### 2.3. Statistical analysis

Analysis was performed using the Statistical Package for Social Sciences (SPSS) v22.0 for Windows (SPSS Inc., Chicago, IL, United States). Results are presented for continuous variables as mean ± standard deviation or as median ± interquartile range for non-normal variables and for qualitative variables as frequencies. Differences between the two groups were evaluated by the *t*-test for parametric and the Mann-Whitney U test for non-parametric variables. Pearson’s or Spearman’s rank tests were performed for univariate comparisons of continuous variables and the Chi-Square Test for qualitative variables. Logarithmic transformation was used when indicated for non-normally distributed data. When logarithmic transformation did not result in normal distributions, non-parametric tests were performed. The Kaplan-Meier method was used for survival analysis, and survival curves of the two groups were compared, using a log-rank test. Cox regression analysis was performed for univariate and multivariate predictors of survival. Considering multicollinearity issues, factors with a significant univariate association were inserted into the multivariate analysis.

## 3. Results

### 3.1. Baseline characteristics

**Figure 1:**
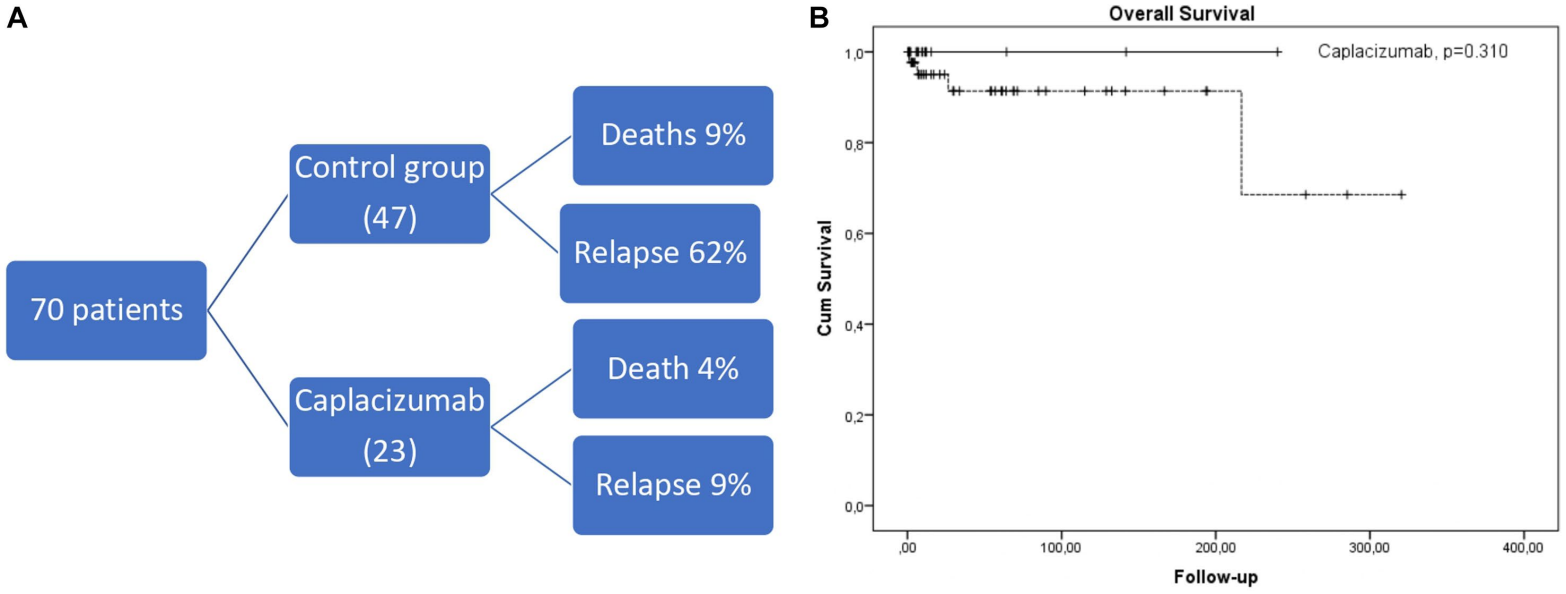
**(A)** Short diagram of patients included and outcomes. **(B)** Kaplan-Meier curve depicting overall survival in caplacizumab (black line) compared to historical control group (dotted line).

We have studied 70 consecutive white patients in total (median age 45 years, range 19–85), of whom 23 were enrolled in the caplacizumab group and the remaining 47 in the historic control group (Figure 1A). Baseline characteristics are presented in Table 1. No significant differences were observed in patient or disease characteristics between the two groups. Comorbidities refer only to clinically significant underlying diseases before TTP diagnosis, such as diabetes, connective tissue disorders. The difference was not meaningfully significant and therefore, no safe conclusions can be made due to the limited sample size.

**Table 1:** Baseline patient characteristics in both groups.

Characteristics	Caplacizumab (*n* = 23)	Historical group (*n* = 47)	All patients (*n* = 70)	*p*-value
**Age (year)**				
Median	47	45	46	0.231
Range, IQR	24–74, 20	19–85, 19	19–85, 17.1	
Female gender (%)	65%	71%	66%	0.485
**BMI (kg/m^2^)**				
Median	27	28	27	0.563
Range, IQR	22–33, 4.1	23–31, 3.5	22–33, 4.2	
**Hb at diagnosis (gr/dL)**				
Median	8.7	9.5	9.0	0.342
Range, IQR	5.3–14.3, 2.8	5.6–13.4, 3.2	5.4–14.3, 3.2	
**Plt at diagnosis (plt/μL)**				
Median	23,000	21,000	22,000	0.763
Range, IQR	3,000–115,000, 15,719	5,000–100,000, 2,300	3,000–115,000, 18,500	
**MCV at diagnosis**				
Median	94.7	88.9	93.2	0.231
Range, IQR	62.6–105, 20.1	80–109; 12.3	62.6–109, 14.2	
**Schistocytes (%)**				
Median	12.3	7.3	11.2	0.654
Range, IQR	3–22, 3.2	1–18, 3.8	1–22, 3.4	
**Creatinine (mg/dL)**				
Median	1.02	1.16	1.05	0.112
Range, IQR	0.7–1.99; 0.4	0.6–3, 0.5	0.6–3, 0.5	
**Fibrinogen (gr/L)**				
Median	1.3	2.7	1.8	0.423
Range, IQR	1.7–5.31, 1.9	1.2–7.6, 2.1	1.2–7.6, 2.0	
**LDH (mg/dL)**				
Median	831	753	794	0.312
Range, IQR	549–1,109, 235	621–1,243, 543	549–1,243, 425	
**Glasgow coma scale**				
Median	13	12	13	0.129
Range, IQR	12–15, 1.2	8–15, 2.1	8–15, 2.1	
Elevated troponin, % *	31%	43%	37%	0.182
**Comorbidities, %**	30%	6%	23%	0.102
**CNS involvement, %**	50%	53%	50%	0.763
**Corticosteroids, %**	100%	100%	100%	NA
**Median TPE, range, IQR (n)**	12, 8–23, 8.6	14, 6–32, 10	12, 6–32, 10	0.231
**Refractory to TPE, %**	19%	44%	24%	0.184
**Rituximab doses, %**	4–9, 79%	2–8, 64%	2–9, 72%	0.287

No significant differences observed between caplacizumab treated and historical control group patients. IQR, interquartile range; BMI, body mass index; Hb, hemoglobin; Plt, platelets; MCV, mean corpuscular volume; LDH, lactate dehydrogenase (upper limit of normal reference range 245 mg/dL); CNS, central nervous system; TPE, therapeutic plasma exchange; NA, not applicable. *Elevated troponin was defined as above the local upper limit of normal reference range.

### 3.2. Treatment modalities

Plasma exchange was applied in all episodes, except for two patients that denied plasma exchange, one in the caplacizumab and one in the historic control group; both with favorable outcomes. All patients received corticosteroids, and the majority of them also received rituximab in both groups, as shown in Table 1. Rituximab as first-line treatment (day 1) was more common in the caplacizumab group compared to historic control (68% versus 32%, *p* < 0.001), possibly reflecting current international guidelines recommendations. Low-dose aspirin (100 mg) was given in all patients and low-dose molecular heparin was started after the increase of platelets at levels above 50,000/μL.

Caplacizumab (10 mg daily) was given at a median on day 7 (1–43) following initial diagnosis for 32 (6–47) dosages. The drug was provided through regular market access. The majority of patients received caplacizumab at the first iTTP episode (15/23; 65%). All patients intended to receive caplacizumab as first-line treatment, except four patients, who received caplacizumab after being unresponsive to plasma exchange (median day 18, range 8-43 from diagnosis). Administration of caplacizumab within 72 hours from diagnosis was achieved in most cases (16/23; 70%), while 3/23 were delayed due to access issues (median day 6, range 4-8 from diagnosis). In two cases, iTTP occurred early after COVID-19 infection. Median plasma exchange sessions were 12 (8–23) in the caplacizumab group versus 14 (6–32) in the control group.

### 3.3. Safety

Caplacizumab administration did not produce any grade 3 complications or major bleeding events. Minor bleeding events were reported in 21% patients that received caplacizumab. and 15% of historic control group patients.

### 3.4. Outcomes

All patients achieved early platelet normalization 5 (3–7) days from caplacizumab administration. In all caplacizumab-treated patients, ADAMTS13 levels became detectable (25–52%) by end of treatment (6–47 doses). No significant difference was found in platelet recovery or plasma exchange duration based upon timing of caplacizumab initiation: earlier initiation (within <72 h of diagnosis; *n* = 16) vs later initiation (>72 hours; *n* = 7).

No exacerbation was observed in the caplacizumab setting. 2/23 patients treated in the caplacizumab group (that also had higher rates of first-line rituximab administration) relapsed within 13 and 18 months of their first caplacizumab dose, with low ADAMTS13 activity (<1%), compared to 29/47 (62%) patients from the historic control group (*p* < 0.001). The first patient re-received caplacizumab at regular doses from day 1 of relapse with adjunctive steroids but without plasma exchange. The second patient received caplacizumab 10 days after the initial relapse because of poor response to plasma exchange and steroids. Both patients’ platelet count normalized rapidly within 10 and 4 days, respectively, from the start of caplacizumab. After a median of 19.0 (2.6–320) months since the iTTP diagnosis, 5 deaths occurred (4 in the control group and 1 in the caplacizumab group, *p* = 0.310, Figure 1B). All deaths were associated with an acute episode, except for one attributed to acute ischemic stroke during remission in the control cohort.

## 4. Discussion

Our real-world multicenter comparative study yields updated information, and it also shows that caplacizumab is safe and effective in treating iTTP, including cases refractory to plasma exchange, re-administration, and cases without previous plasma exchange treatment. Patients treated with caplacizumab in this study had no major hemorrhagic events or other complications. In addition, cessation of dosing guided by ADAMTS13 has ensured a low relapse rate. Despite early diagnosis and the drug’s wide availability, our real-world data confirm that treatment initiation is only sometimes feasible from the day of initial diagnosis.

The latter might account for the lack of statistically significant differences between the control group, and the rather small patient population. This is implied by a recent real-world patient experience, confirming that early initiation confers better outcomes superior to those reported by historic controls (12–14). Therefore, we have summarized available evidence of real-world studies comparing caplacizumab to a control group in Table 2. However, early initiation is not always feasible in the real-world setting, not only due to drug availability issues, but also because ADAMTS13 testing cannot be readily available before the first plasma exchange. Trying to overcome this issue, ISTH guidelines have introduced risk scores, to predict ADAMTS13 activity, which have high accuracy in all patients, except for those with secondary causes. It should be also noted that secondary causes cannot be always recognized immediately, before the first plasma exchange. An additional important component that is neglected by many studies is the importance of ADAMTS13 level measurement in caplacizumab dosing. Furthermore, as the patient population that has received caplacizumab continues to grow, additional questions arise, regarding re-administration and plasma exchange-free treatment approach. Beyond caplacizumab, differences in studies with historical controls need to take into account the increasing use of rituximab over the last years, in accordance with ISTH guidelines. Lastly, high cost remains a challenge to its widespread use until clear evidence is provided for plasma exchange-free treatment (15).

**Table 2:** Summary of real-world data comparing outcomes in caplacizumab-treated versus historical control patients.

Country (year)	Centers	Control group	Treatment	Patient number	% presenting with recurrent TTP	% with ADAMTS13 <10%
France (2021) (11)	Multicenter	Historical	Caplacizumab + SOC	90	13	100
			SOC	180	12	100
UK (2021) (15)	Multicenter	Historical	Caplacizumab + SOC	85	NA	99
			SOC	39	NA	NA
Spain (2022) (16)	Multicenter	Concurrent	Caplacizumab + SOC	77	4.5	100
			SOC	78	20.5	100
Germany/Austria (2023)	Multicenter	Historical	Caplacizumab + SOC	113	2	97
			SOC	119	4	92

UK, United Kingdom; SOC, standard of care; No, number; TTP, thrombotic thrombocytopenic purpura; NA, not available; ADAMTS13, a disintegrin and metalloprotease with thrombospondin type 1 repeats, member 13.

Real-world evidence is of utmost importance in rare diseases, like iTTP (13, 17). This study is a multicenter collection of real-world data, presenting the use of caplacizumab outside of clinical trials. Further patient recruitment is necessary to provide additional data. Given that this is indeed a rare but life-threatening disease, we want to emphasize on the safety and efficacy that caplacizumab has brought not only to the acute setting but also to the relapsed setting. A unique characteristic of our study was that cessation of caplacizumab treatment was based on ADAMTS13 activity, leading to low relapse rates with daily dosing, as noted by Tse et al. (18). Alternate dosing of caplacizumab has been also suggested by the German group, with an individualized algorithm (19). In context with TITAN and HERCULES (7, 8), an important aspect of our trial is also the low number of deaths (only 1) in the caplacizumab group. Deaths in the control group were 4 in our study, possibly due to the long-term follow-up of historical controls.

In conclusion, our study confirms the safety of caplacizumab in treating iTTP. While overall TPE durations did not differ between groups, the addition of caplacizumab was associated with rapid platelet recovery and low relapse rates. Given the limited international clinical experience with caplacizumab, dosing modifications, compared to the clinical trial setting, have non-inferior outcomes in the real-world setting (16, 19). Since ADAMTS13 reduction has emerged as an essential indicator of long-term results, further studies in large real-world populations with longer follow-ups are needed to delineate the iTTP treatment algorithm in the era of personalized medicine.

## Data availability statement

The raw data supporting the conclusions of this article will be made available by the authors, without undue reservation.

## Ethics statement

The studies involving humans were approved by General Hospital of Thessaloniki “G. Papanikolaou.” The studies were conducted in accordance with the local legislation and institutional requirements. The participants provided their written informed consent to participate in this study.

## Author contributions

EG: Conceptualization, Data curation, Methodology, Project administration, Supervision, Visualization, Writing – original draft. EN: Conceptualization, Methodology, Project administration, Validation, Writing – original draft. EE-K: Methodology. SD-B: Methodology. ChaK: Methodology. AP: Methodology. AM: Methodology. ChaP: Methodology. ChrK: Methodology. AB: Methodology. IT: Methodology. TT: Methodology. TC: Methodology. MP: Methodology. AntS: Methodology. EZ: Methodology. GK: Methodology. IS: Visualization, Writing – review & editing. EM: Writing – review & editing. ZM: Writing – review & editing. DK: Writing – review & editing. AK: Writing – review & editing. ArgS: Writing – review & editing. EK: Writing – review & editing. HP: Writing – review & editing. CL: Writing – review & editing.
